# Meetings of Societies

**Published:** 1951-10

**Authors:** 


### Meetings of Societies

Bristol Medico-Chirurgical Society

Programme for 1951–52

10th October. Annual General Meeting : President's Address.

14th November. Clinical Meeting.

12th December. **" Hiatus Hernia "**, Mr. Ronald Belsey.

*Editor's Report*

As the balance sheet shows, there has been a slight increase in the cost of production this year, and a slight fall in receipts from advertisements. On the other hand, we have received some arrears of subscriptions from non-members, as well as an increase in subscriptions for the year, and the financial position appears reasonably sound. As a matter of interest we may compare the cost of production of four issues in various years:

| | |
|---|---|
| 1898 | £207 |
| 1914 | £144 |
| 1919 | £248 |
| 1947 | £287 |
| 1951 | £603 |

But it is clear that we cannot relax any of the restrictions recently adopted. Indeed we have had this year to decide that we can no longer publish obituary notices: we are becoming an older journal, so must expect an

### BRISTOL MEDICO-CHIRURGICAL SOCIETY

### INCOME AND EXPENDITURE ACCOUNT for eleven months ENDED 31st AUGUST, 1951

| EXPENDITURE | £ | s. | d. | INCOME | £ | s. | d. |
|---|---|---|---|---|---|---|---|
| To Subscriptions to *Medico-Chirurgical Journal* ... ... ... ... ... | 316 | 0 | 0 | By Members' Subscriptions (395 at £1 11s. 6d.) ... ... ... ... | 622 | 2 | 6 |
| ,, Grant to Medical Library ... ... | 25 | 0 | 0 | ,, Interest on Bank Deposit Account ... | | 12 | 9 |
| ,, Lewis's Medical and Scientific Library ... | 7 | 11 | 8 | | | | |
| ,, Librarian, Honorarium ... ... ... | 15 | 0 | 0 | | | | |
| ,, Treasurer's Secretary ... ... ... | 10 | 0 | 0 | | | | |
| ,, Transport of Patients ... ... ... | 10 | 18 | 9 | | | | |
| ,, Expenses *re* Meetings ... ... ... | 7 | 7 | 0 | | | | |
| ,, Audit Fee ... ... ... ... ... | 10 | 10 | 0 | | | | |
| , Printing and Stationery ... ... ... | 31 | 5 | 11 | | | | |
| ,, Catering ... ... ... ... ... | 71 | 17 | 6 | | | | |
| ,, Postages and Sundries ... ... ... | 18 | 6 | 11 | | | | |
| ,, Wreath (Professor Nixon) ... ... | 5 | 0 | 0 | | | | |
| | £528 | 17 | 9 | | | | |
| ,, Surplus for 1951 ... ... ... ... | 93 | 17 | 6 | | | | |
| | £622 | 15 | 3 | | £622 | 15 | 3 |

### BALANCE SHEET AS AT 31st AUGUST, 1951

| LIABILITIES | £ | s. | d. | £ | s. | d. | ASSETS | £ | s. | d. | £ | s. | d. |
|---|---|---|---|---|---|---|---|---|---|---|---|---|---|
| | | | | | | | Cash at Bank: | | | | | | |
| Sundry Creditors ... ... ... ... | | | | 101 | 1 | 8 | Current Account ... ... | 270 | 9 | 11 | | | |
| Cash due to the *Journal* ... ... ... | | | | 153 | 4 | 9 | Deposit Account ... ... | 174 | 8 | 5 | | | |
| Accumulated Fund as at 30th September, 1950 ... ... | 258 | 15 | 4 | | | | | | | | 444 | 18 | 4 |
| *Deduct:* Outstanding subscriptions written off ... ... | 78 | 13 | 6 | | | | Petty Cash in hands of Librarian ... ... | | | | | 3 | 5 |
| | 180 | 1 | 10 | | | | Arrears of Subscriptions ... ... ... | | | | 83 | 4 | 0 |
| *Add:* Surplus for 1951 ... | 93 | 17 | 6 | | | | | | | | | | |
| Accumulated Fund, August, 1951 | | | | 273 | 19 | 4 | | | | | | | |
| | | | | £528 | 5 | 9 | | | | | £528 | 5 | 9 |

13 Meadow Street, Weston-super-Mare,
*August 23rd*, 1951.

Audited and found correct.
C. J. RYLANDS & CO., *Chartered Accountants.*

### BRISTOL MEDICO-CHIRURGICAL JOURNAL

### INCOME AND EXPENDITURE ACCOUNT for eleven months ENDED 31st, AUGUST 1951

### EXPENDITURE

| | | £ s. d. | £ s. d. |
|---|---|---|---|
| Arrowsmith, Printing: | | | |
| January, 1951 ... ... | | 165 4 4 | |
| April ... ... ... ... | | 164 5 4 | |
| July ... ... ... ... | | 133 6 5 | |
| October (estimate) ... ... | | 165 0 0 | |
| | | 627 16 1 | |
| October, 1950: | | | |
| Estimate ... | £150 0 0 | | |
| Cost ... ... | 125 10 8 | | |
| *Deduct* ... ... ... | | 24 9 4 | |
| | | 603 6 9 | |
| Wrappers, etc. ... ... | | 12 18 4 | |
| | | | 616 5 1 |
| Postages and Sundries ... ... ... ... | | | 7 4 3 |
| Audit Fee ... ... ... ... ... | | | 4 4 0 |
| | | | 627 13 4 |
| Surplus for 1951 ... ... ... ... | | | 8 17 10 |
| | | | £636 11 2 |

### INCOME

| | | £ s. d. | £ s. d. |
|---|---|---|---|
| Subscriptions: | | | |
| 395 Members of the Society at 16s.* | | | 316 0 0 |
| 45 Non-Members ... ... ... ... | | | 36 0 0 |
| Arrears (non-members) ... ... ... | | | 24 2 6 |
| Miscellaneous Sales, 1945–1950 ... ... | | | 34 8 6 |
| Advertisements: | | | |
| January, 1951 ... ... | | 56 17 10 | |
| April ... ... ... ... | | 56 12 4 | |
| July ... ... ... ... | | 57 16 0 | |
| October (estimate) ... ... | | 50 0 0 | |
| October, 1950 | 54 14 0 | | |
| *Less* estimate | 50 0 0 | | |
| | | 4 14 0 | |
| | | | 226 0 2 |
| | | | £636 11 2 |

### BALANCE SHEET AS AT 31st AUGUST, 1951

### LIABILITIES

| | | £ s. d. | £ s. d. |
|---|---|---|---|
| Arrowsmith, for Printing: | | | |
| Due September, 1950 ... | | 494 6 10 | |
| For 1951 ... ... ... ... | | 616 5 1 | |
| | | 1,110 11 11 | |
| Advertisements | £226 0 2 | | |
| Miscellaneous Sales | 34 8 6 | | |
| Cash Paid ... | 659 12 10 | | |
| Due August, 1951 | | 920 1 6 | |
| | | | 190 10 5 |
| Auditors ... ... ... ... ... | | | 4 4 0 |
| | | | 194 14 5 |
| Surplus for 1951 ... ... | | 8 17 10 | |
| Surplus September, 1950 ... | | 162 14 3 | |
| Surplus August, 1951 ... ... | | | 171 12 1 |
| | | | £366 6 6 |

### ASSETS

| | £ s. d. |
|---|---|
| Cash at Bank ... ... ... ... ... | 213 1 9 |
| Cash due from Bristol Med.-Chirurgical Society* (included in sum of £316 0 0 above) ... ... ... ... ... | 153 4 9 |
| | £366 6 6 |

Audited and found correct.
C. J. RYLANDS & CO., *Chartered Accountants.*
13 Meadow Street, Weston-super-Mare,
*August 23rd*, 1951.

* Although only 395 subscriptions have been received, the *Journal* was sent to 404 members in 1951. Thus there is also due to the *Journal* for 1951 the sum of £7 4s. od., added to £7 4s. od. for 1950 (*Jl.* LXVIII, p. 29), a total of £14 8s. od.

increase in the number of former subscribers for whom such notices would be required—and a notice restricted to a mere few lines is obviously unsatisfactory. We seem now to have reached the irreducible minimum and must hope that no further sacrifice will be needed. We have decided that it should be possible to afford to print the " List of Subscribers " every third year. By omitting degrees and addresses the cost is about halved : this year it amounted to £20.

The number of journals received in exchange for our own is ninety-three, priced at £120. This is a contribution from subscribers to the *Journal* to the University Medical Library.

### Plymouth Medical Society

Programme 1951–52

3rd October. Annual General Meeting.

17th October. Dance.

19th October. **" The Medical Witness in Crimes of Violence ",** Dr. F. E. Camps.

16th November. **" Intestinal Obstruction ",** Professor Aird.

January, 1952. **" Peripheral Vascular Disease",** Mr. Boyd.

### Torquay and District Medical Society

*President:* Dr. Colin MacVicker. *Hon Secretary:* Dr. R. A. Lattey, Ambrook, Rousdown Road, Torquay. *Hon. Treasurer:* Dr. J. R. Imrie.

*Calendar*, 1951–52

(Meetings Torbay Hospital 8.30 p.m.: unless otherwise stated)

1951, 4th October. 4.30 p.m. **" Industrial Medicine ",** Dr. W. J. Lloyd. 7.30 p.m. Dinner and Dance at the Victoria Hotel, Torquay.

25th October. **" Cortisone and ACTH ",** Prof. G. R. Cameron.

8th November. Clinical Discussion.

22nd November. **" Research in Medicine."**

13th December. **" The Treatment of the Elderly ",** Dr. Marjory Warren.

1952, 10th January. 3 p.m. Clinical Meeting with Branch of the B.M.A.

24th January. **" Modern Aspects of Skin Conditions ",** Dr. John Franklin.

14th February. Clinical Discussion.

28th February. **" Difficult Behaviour in Children ",** Dr. R. F. Barbour.

13th March. 4.30 p.m. Newton Abbot Hospital: Clinical Meeting.

27th March. " **Teaching Medicine.**"

24th April. " **Crime Reconstruction** ", Dr. Keith Simpson.

12th June. 3 p.m. Summer Meeting: Royal Naval College, Dartmouth.

### South Wales Society of Anaesthetists

A meeting of this Society was held at the Royal Gwent Hospital on Saturday, 30th June, 1951. Demonstrations were given in the morning and in the afternoon. Dr. Musgrove (Cardiff) spoke on " **Hypotension** " and Dr. Rendall Baker (Cardiff) on " **Anaesthesia in Sweden** ".

### Welsh Paediatric Club

A meeting of this Club was held at the Royal Gwent Hospital, Newport, on 28th February, 1951: the President, Professor A. G. Watkins, in the chair. The first part of the afternoon was devoted to a clinical meeting, various members demonstrating in all some twenty rare and interesting conditions. This meeting was followed by a discussion and then a film demonstration of Rammstedt's operation by Mr. J. T. Rice-Edwards.

### West Somerset Medical Club

*President:* J. F. Coates. *Treasurer:* J. M. Thomson. *Secretary:* C. E. M. Ware.

Meetings first Thursday in each month.

October. " **Animal Diseases Communicable to Man** ", Professor Blakemore.

### British Medical Association

### Newport Division

During the past year the following programme has been carried out.

October, 1950.—Lecture Demonstration on " **Hypnotism** " by Dr. George King.

November, 1950.—Discussion on " **Peptic Ulceration** " by Mr. Scanlon, Dr. Grahame Jones, Mr. Rice-Edwards and Dr. Wade Thomas.

January, 1951.—Discussion on " **Medicine and the Welfare State** ".

February, 1951.—A joint clinical meeting with the South Wales Branch at the new Plastic Unit, Chepstow. Demonstrations by Mr. Emlyn Lewis.

March, 1951.—Annual B.M.A. Lecture " **Legal Hazards in Medical Practice** " by Dr. Forbes, Secretary of Medical Defence Union.

May, 1951.—" **The Extremes of Life.**" Joint address given by Dr. T. A. Brand and Dr. Morag Insley.

